# Rapid Generation of Human-Like Neutralizing Monoclonal Antibodies in Urgent Preparedness for Influenza Pandemics and Virulent Infectious Diseases

**DOI:** 10.1371/journal.pone.0066276

**Published:** 2013-06-18

**Authors:** Weixu Meng, Weiqi Pan, Anna J. X. Zhang, Zhengfeng Li, Guowei Wei, Liqiang Feng, Zhenyuan Dong, Chufang Li, Xiangjing Hu, Caijun Sun, Qinfang Luo, Kwok-Yung Yuen, Nanshan Zhong, Ling Chen

**Affiliations:** 1 State Key Laboratory of Respiratory Disease, Guangzhou Institute of Biomedicine and Health, Chinese Academy of Sciences, Guangzhou, China; 2 The First Affiliated Hospital of Guangzhou Medical University, Guangzhou, China; 3 Department of Microbiology, State Key Laboratory of Emerging Infectious Diseases, The University of Hong Kong, Hong Kong, China; Shanghai Medical College, Fudan University, China

## Abstract

**Background:**

The outbreaks of emerging infectious diseases caused by pathogens such as SARS coronavirus, H5N1, H1N1, and recently H7N9 influenza viruses, have been associated with significant mortality and morbidity in humans. Neutralizing antibodies from individuals who have recovered from an infection confer therapeutic protection to others infected with the same pathogen. However, survivors may not always be available for providing plasma or for the cloning of monoclonal antibodies (mAbs).

**Methodology/Principal Findings:**

The genome and the immunoglobulin genes in rhesus macaques and humans are highly homologous; therefore, we investigated whether neutralizing mAbs that are highly homologous to those of humans (human-like) could be generated. Using the H5N1 influenza virus as a model, we first immunized rhesus macaques with recombinant adenoviruses carrying a synthetic gene encoding hemagglutinin (HA). Following screening an antibody phage display library derived from the B cells of immunized monkeys, we cloned selected macaque immunoglobulin heavy chain and light chain variable regions into the human IgG constant region, which generated human-macaque chimeric mAbs exhibiting over 97% homology to human antibodies. Selected mAbs demonstrated potent neutralizing activities against three clades (0, 1, 2) of the H5N1 influenza viruses. The *in vivo* protection experiments demonstrated that the mAbs effectively protected the mice even when administered up to 3 days after infection with H5N1 influenza virus. In particular, mAb 4E6 demonstrated sub-picomolar binding affinity to HA and superior *in vivo* protection efficacy without the loss of body weight and obvious lung damage. The analysis of the 4E6 escape mutants demonstrated that the 4E6 antibody bound to a conserved epitope region containing two amino acids on the globular head of HA.

**Conclusions/Significance:**

Our study demonstrated the generation of neutralizing mAbs for potential application in humans in urgent preparedness against outbreaks of new influenza infections or other virulent infectious diseases.

## Introduction

Outbreaks of infectious diseases, such as the severe acute respiratory syndrome (SARS) epidemic in 2003 and several influenza pandemics especially H5N1, H1N1, and most recently the emergent cases of H7N9, have caused loss of human life, public panic, and economic setbacks. Vaccines against specific pathogens are the most effective means of protecting humans from infection. However, it takes many years or even decades, to research, develop and manufacture a vaccine against an emerging pathogen. Preparedness for new pandemics or outbreaks of virulent infectious diseases has been a challenging demand on public health. It has been shown that individuals who recover from H5N1 or H1N1 viral infections can generate neutralizing antibodies against the pathogen, and their plasma confers therapeutic protection in infected individuals when administered passively [1,2]. However, plasma from convalescent individuals may not be available in sufficient quantities or may be nonexistent if there are no survivors in future pandemics or new and emerging infectious diseases. Therefore, a method to rapidly generate and select neutralizing antibodies is urgently needed for the defense against new virulent pathogens.

Although monoclonal antibodies can be generated in mice immunized with a specific antigen through hybridoma technology, the immunogenicity of non-human antibodies requires humanization, which is a prolonged and labor-intensive process. The screening of a naive or synthetic antibody phage display library of human origin can lead to the identification of human-like mAbs; however, there are concerns regarding the lack of *in vivo* maturation against the target antigen to obtain the optimal neutralizing antibodies exhibiting high affinity and potency. Several methods have been reported in which neutralizing mAbs have been cloned from infected or vaccinated individuals using single B cell cloning or phage display [3-13]. However, a human survivor may not be available during every outbreak, and there are ethical and legal issues associated with using human subjects for immunizing an individual with a pathogen or antigen, especially when an approved vaccine is not available. Because the genome and immunoglobulin genes in rhesus macaques share over 92% homology with humans [14,15], we generated human-like mAbs from rhesus macaques immunized with target antigens.

In this study, we used the influenza virus as a model pathogen to demonstrate an integrated solution to generate high affinity neutralizing mAbs that were around 97% identical to human immunoglobulin. The highly pathogenic avian influenza virus H5N1 exhibits a high mortality rate in humans [16]. Given the absence of anti-H5N1 immunity in the human population, there are concerns about the possibility of a catastrophic influenza pandemic should a H5N1 virus gain human-to-human transmission ability. The two classes of antiviral drugs that have been used for early intervention in treating influenza A viral infection are 1) the neuraminidase (NA) inhibitors oseltamivir and zanamivir, which inhibit viral maturation and spreading and 2) the M2 ion channel blockers amantadine and rimantadine, which block the viral M2 proton channel and inhibit viral uncoating after entry. However, these drugs are only effective when used during the early stage of infection, and there are increasing reports of resistance to these drugs [17]. Studies using mouse models and clinical observations have indicated that neutralizing antibodies may provide better therapeutic efficacy than existing antiviral drugs, even at the later stages of infection [1,2,18]. In this study, we demonstrated an integrated process as follows: 1) the use of recombinant adenoviral vectors carrying synthetic genes encoding the target antigens allowed the rapid preparation of antigens without the need for having the pathogen in hand; 2) the immunization of rhesus macaques (*Macaca Mulata*) with the vectored antigen or the pathogen, if available; 3) the screening of an antibody phage display library derived from B cells harvested from immunized macaques; 4) cloning the macaque immunoglobulin heavy chain and light chain variable regions and combining them with human constant regions to generate human-macaque chimeric mAbs; and 5) further characterization of the mAbs using *in vitro* and *in vivo* studies. Our study demonstrated a practical option for the rapid generation of neutralizing mAbs for potential application in humans in preparedness for outbreaks of a new influenza or other virulent infectious diseases. 

## Results

### Immunization with adenovirus carrying codon-optimized genes encoding HA induced neutralizing antibodies against H5N1 influenza viruses in rhesus macaques

**Figure 1:**
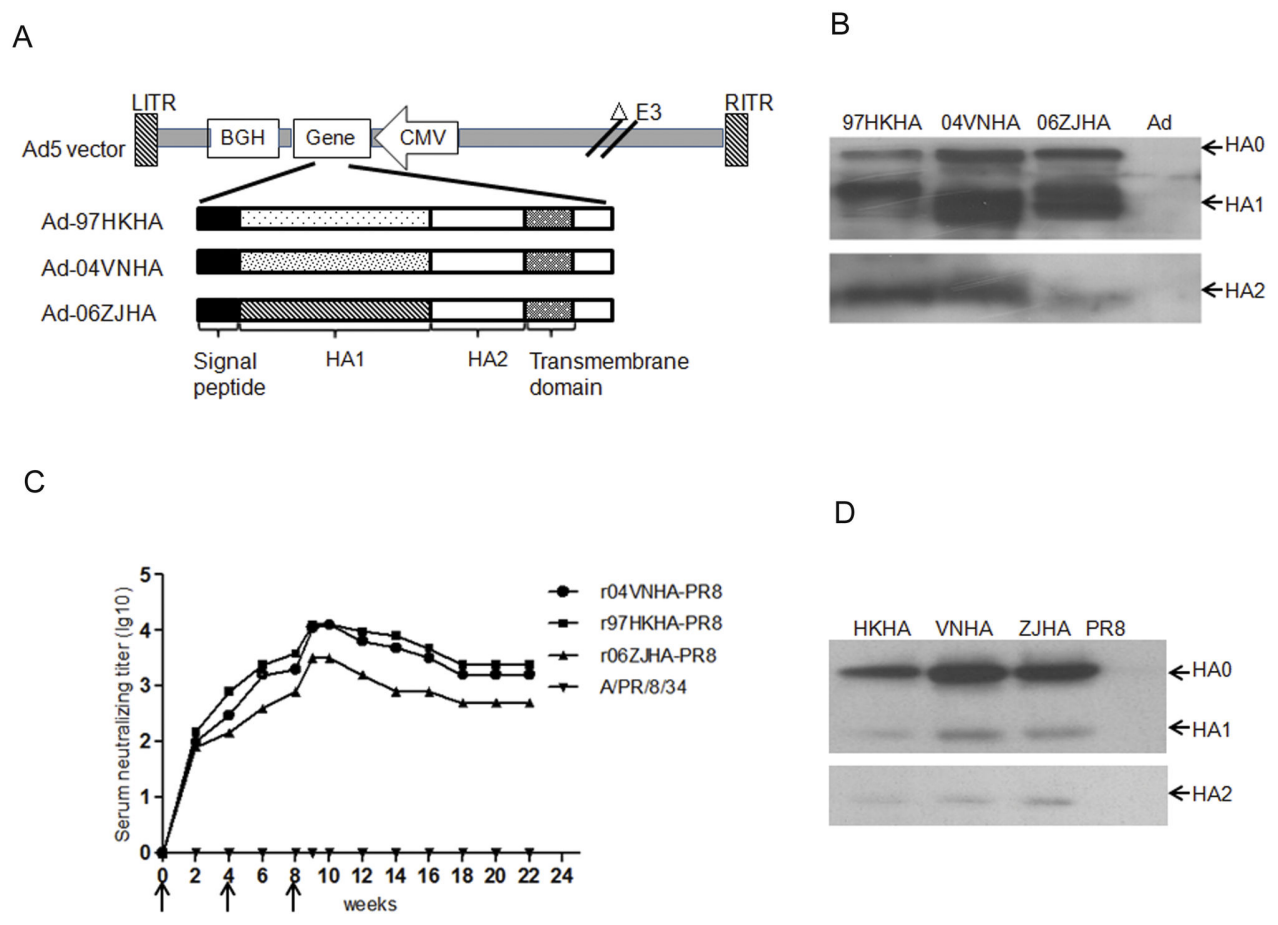
Recombinant adenoviruses carrying codon-optimized HA genes expressed HA proteins and elicited neutralizing antibodies against H5N1 viruses in rhesus macaques. (A) Schematic representation of adenoviral vectors carrying H5N1 influenza HA gene (Ad5-HA). The expression cassette, containing the HA gene under the control of the CMV promoter and BGH polyA signal, was cloned into the adenoviral E1 region. (B) Expression of the HA protein mediated by recombinant adenovirus Ad5-HAs. The HA proteins before cleavage (H0) and after cleavage (HA1 and HA2) were detected using chicken serum against HA in Western blots. The Vero cells were harvested for analysis at 48 hours after infection with Ad-Empty, Ad5-HA04VN, Ad5-HA97HK, and Ad5-HA06ZJ at MOIs of 10. (C) Dynamics of the antibody response in a Chinese rhesus macaque immunized with Ad5-HAs. The sera were collected at 2-week intervals. The neutralizing titers against the recombinant H5N1 viruses (r97HKHA-PR8, r04VNHA-PR8, and r06ZJHA-PR8) were determined using a micro-neutralization assay. The arrows indicate the time of immunization. (D) The binding specificity of the sera against HA of different clades expressed by the recombinant influenza viruses was determined by Western blot analysis.

In general, immunization requires the collection and manipulation of the target pathogen to prepare related antigens for immunization. During a pandemic, the pathogen may not be available because of bio-safety, transportation, and technology, issues and even social or political barriers. Earlier studies by our lab and others [19-21] have shown that the adenovirus-mediated delivery of antigens, including influenza HA, induces a robust antibody response within a short period after immunization. In this study, we first synthesized three hemagglutinin (HA) genes based on human codon usage for optimal expression in mammalian cells. Next, we constructed three recombinant replication-incompetent adenoviruses (Ad5), Ad5-HA(97HK), Ad5-HA(04VN), and Ad5-HA(06ZJ), carrying the full-length HA genes representing three different clades of H5N1 viruses, including the HAs of A/Hong Kong/482/97 (clade 0), A/Vietnam/1194/04 (clade 1), and A/Zhe Jiang/16/06 (clade 2), respectively. The adenoviral vector-mediated expression of the HA proteins was confirmed using Western blot analysis of Ad5-HA-infected HEK293 cell lysates, which revealed the expression of HA0 and its cleavage into HA1 and HA2 (Figure 1a).

We immunized two rhesus macaques with a mixture of 1x10^11^ viral particles of each Ad5-HA at weeks 0, 4, and 8 via intramuscular injection. At various intervals, the serum samples were collected for the detection of neutralizing antibodies against the different clades of H5N1 viruses. A micro-neutralization (MN) assay was used to monitor the kinetics of the neutralizing antibodies against recombinant influenza viruses carrying each of the three HA clades in the PR8 backbone, r97HKHA-PR8, r04VNHA-PR8, and r06ZJHA-PR8 influenza viruses, respectively. The neutralizing antibodies were detected at 2 weeks after the initial immunization, the levels were increased at 4 weeks and continued to increase at 6 weeks and 10 weeks following additional immunizations. The neutralizing antibodies were maintained at high levels for at least 22 weeks. The sera collected from the macaques from 2 to 22 weeks neutralized all three clades of the H5N1 recombinant viruses but not the H1N1 influenza virus PR8 (Figure 1b). The Western blot analysis confirmed the presence of serum antibodies that bound the HA0, HA1, and HA2 of all three clades of the H5N1 viruses tested but not the H1N1 virus PR8 (Figure 1c). These data demonstrated that the adenovirus-mediated expression of HA effectively elicited the production of neutralizing antibodies against HA proteins in rhesus macaques as early as two weeks after immunization.

### Broadly neutralizing mAbs to H5N1 influenza viruses are readily generated from an immunized antibody phage library

**Figure 2:**
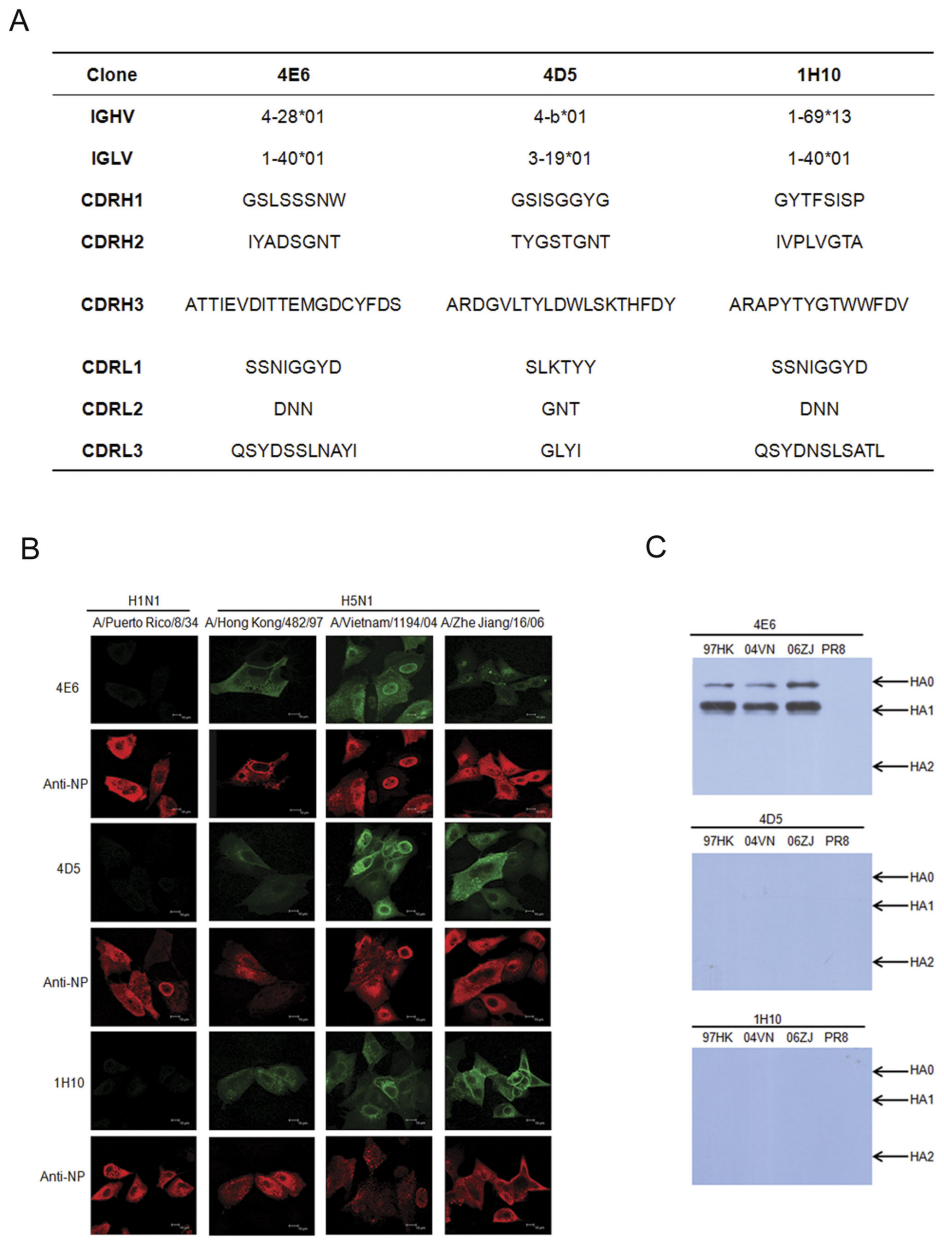
Analysis of mAbs against HAs of clade 0, 1, and 2 H5N1 viruses. (A) Amino acid sequences of VH and VL region and their closest corresponding human Ig gene. (B) Immunofluorescent analysis of mAbs. The MDCK cells were infected with H1N1 influenza virus A/Puerto Rico/8/34 (PR8) or recombinant influenza viruses carrying HA from clade 0, 1, and 2 H5N1 viruses in r97HKHA-PR8, r04VNHA-PR8, and r06ZJHA-PR8. (C) Western blot analysis. The HA proteins expressed in MDCK cells infected with r97HKHA-PR8, r04VNHA-PR8, r06ZJHA-PR8 or A/Puerto Rico/8/34 were used to determine the binding of the mAbs. Arrows indicate the specific bands for the corresponding proteins.

The mRNA was extracted from the peripheral blood mononuclear cells (PBMCs) of immunized macaques and was used to construct an antibody phage library displaying single-chain variable fragments (scFvs) containing the immunoglobulin variable regions of heavy chain (VH) and light chain (VL). Based on published data regarding the rhesus immunoglobulin genes and the closest corresponding human immunoglobulin genes [22-31], we used PCR to amplify the VH and the VL cDNAs derived from B cells. PCR products were used to construct scFv-presenting phage libraries for each macaque. Each library contained approximately 2.4 x 10^7^ clones. The phage library from one of the macaques was subjected to 2 rounds of panning using a recombinant VN04HA protein as the target antigen. The ELISA screening using inactivated recombinant influenza viruses resulted in the isolation of 87 clones. Based on the analysis of the VH sequences, 39 scFv clones containing unique complementarity-determining region (CDR) sequences were selected for conversion into full-length IgG1 antibodies by cloning into the gene encoding the human constant region (IgG1) for expression in mammalian cells. The human-macaque chimeric mAbs (8 in total) demonstrated strong neutralizing activities against all three clades of H5N1 viruses using a plaque-reduction assay and the r04VNHA-PR8, r97HKHA-PR8, and r06ZJHA-PR8 viruses. For further analysis, we selected three mAbs that represented different families of VH and VL (Figure 2a). Notably, mAbs 4E6 and 4D5 contained a somewhat longer CDRH3 measuring 19 amino acid residues. The VHs in 4E6 and 4D5 demonstrated the most similarity to the human VH4 family, whereas the VH in 1H10 most closely resembled the human VH1-69 family. The immunofluorescent analysis confirmed the binding of these three mAbs to viruses in cells infected with the three clades of the recombinant H5N1 viruses but did not exhibit reactivity against the H1N1 virus PR8 (Figure 2b). The Western blot analysis demonstrated that mAb 4E6 reacted with the HA0 and HA1 fragments of all three HAs but did not demonstrate reactivity against the HA2 region (Figure 2c). In the Western blot, the mAbs 4D5 and 1H10 did not demonstrate reactivity against HA proteins, indicating that these mAbs likely bind to conformational epitopes on HA.

### Human-macaque chimeric mAbs neutralize H5N1 influenza viruses via different mechanisms of action

The influenza viral HA protein mediates viral entry in two steps. In the attachment step, HA binds to the sialic acid cell surface receptor, and the virus is endocytosed into the cell, and in the post-attachment step, the acidification of the endosome induces a conformational change in HA that leads to the release of viral RNA for subsequent replication. First, we used a micro-neutralization assay to determine the neutralizing activities of these mAbs against the r97HKHA-PR8, r04VNHA-PR8, and r06ZJHA-PR8 recombinant influenza viruses (Table 1). The mAbs demonstrated cross-clade neutralizing activities against these H5N1 viruses but not against the H1N1 virus PR8. The mAb 4E6 exhibited 100% inhibition at a potency of less than 0.39 µg/ml for the clade 0 and the clade 1 H5N1 viruses and at a moderate potency (6.25 µg/ml) against the clade 2 virus. The mAb 1H10 demonstrated 100% inhibition against the clade 0 virus at 1.39 µg/ml and against the clade 1 and clade 2 viruses at 6.25 µg/ml. We then performed a hemagglutination inhibition (HAI) assay for the three mAbs (Table 2), and the results indicated that 4E6 and 4D5 blocked the binding of the virion to the cell surface receptor, whereas 1H10 likely affected the post-attachment process. Taken together with the Western blot and immunofluorescent analyses, the data suggest that that 4E6 likely bound to an epitope on the globular head of HA, whereas 1H10 bound to a conformational epitope that is not associated with cell receptor binding but is important for the post-attachment step.

**Table 1:** Minimum inhibitory concentrations required to neutralize H5N1 influenza viruses.

**Clone**	**IC100 Neutralizing activity**(μ**g/ml**)							
	**r97HKHA-PR8**		**r04VNHA-PR8**		**r06ZJHA-PR8**		**PR8**	
	Clade 0		Clade 1		Clade 2			
**4E6**	<0.39		<0.39		6.25		N	
**4D5**	22.25		13.9		<1.39		N	
**1H10**	<1.56		6.25		6.25		N	

**Table 2:** Hemagglutination inhibition assay (HAI) to measure receptor binding inhibition of mAbs against H5N1 influenza viruses (μg/ml).

	**r97HKHA-PR8 (clade 0)**		**r04VNHA-PR8 (clade 1)**		**r06ZJHA-PR8 (clade 2)**		**PR8**	
**4E6**	0.39		0.19		12.5		N	
**4D5**	>100		25		0.8		N	
**1H10**	>100		>100		>100		N	

### Human-macaque chimeric mAbs bind to HA protein with high affinities and are highly homologous to human antibodies

**Figure 3:**
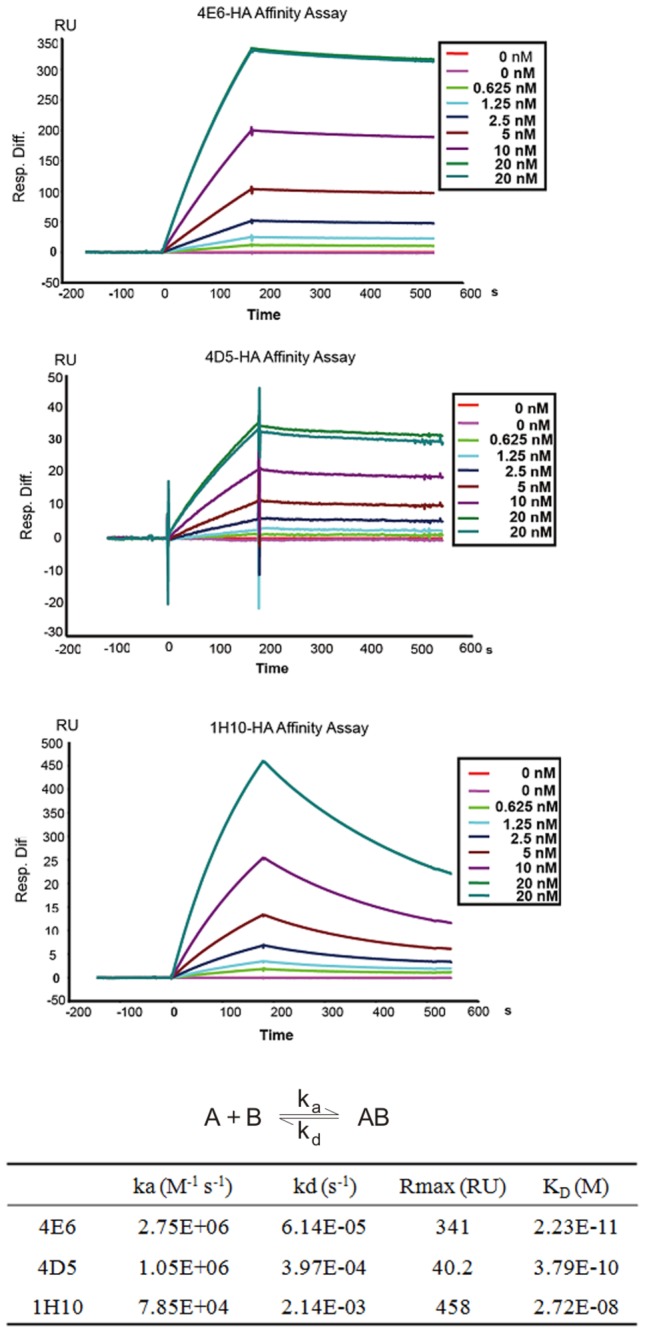
Binding kinetics of mAbs to HA protein.

Next, we measured the affinities of the human-macaque chimeric mAbs to the HA protein in a surface plasmon resonance (SPR) assay using a BIAcore 3000 instrument (Figure 3). In particular, the mAb 4E6 exhibited an extremely high affinity of 22.3 pM and an extremely slow dissociation rate of 6.14x10^-5^/s^-1^ to the HA protein from H5N1 A/Vietnam/1194/04. The mAb 1H10 exhibited a high affinity (27.2 nM) to the HA protein of H5N1 A/Vietnam/1194/04. Both 4E6 and 4D5 mAbs contained a somewhat longer CDR-H3 (19 amino acids), which might have contributed to the high affinity and slow dissociation rate for the HA protein. Because 4E6 exhibited an extremely high affinity to HA and potent neutralizing activities against H5N1, we performed studies to identify the 4E6 binding site on HA. An escape mutant was generated by incubating r04VNHA-PR8 in the presence of a low concentration of 4E6 (Figure S1). Our data demonstrated that the mutations of aspartate (D) to asparagines (N) at amino acid position 183 (D183N) and lysine (K) to glutamate (E) at amino acid position 189 (K189E) on the globular head resulted in the escape from the 4E6 neutralization. Furthermore, this escape mutant showed no response in the HAI to 4E6 at the concentration that could completely block hemagglutination by r04VNHA-PR8. The bioinformatics analysis demonstrated that these two amino acids are highly conserved among the majority of H5N1 viruses. All H5N1 viruses contain D183, most contain K189 or isoelectric R189. Therefore, 4E6 appears to be a broadly neutralizing antibody that may neutralize the majority of H5N1 viruses. Further study will be required to verify if 4E6 neutralizes all H5N1 that contain D183 and K(R) 189.

The association rates (*ka*), dissociation rates (*kd*), and affinity constants (*K*_*D*_) were evaluated using BIACORE 3000 evaluation software using a 1:1 Langmuir binding with a mass transfer model.

To assess the degree of humanness of the mAbs, we analyzed the amino acid sequence in the macaque framework region, which revealed a high degree of homology with the human immunoglobulin gene. Using the germline index (GI) [32] for comparison (http://www.imgt.org/3Dstructure-DB/cgi/DomainGapAlign.cgi), all three mAbs scored within the range for human-derived mAbs. The comparison was made using two human mAbs cloned from individuals infected with influenza virus CR6261 [12] and F10 [9]. The VH region of the human mAbs CR6261 and F10 exhibited a GI value from 84% to 92.75%, whereas the VH region of the macaque mAbs 4E6, 1H10, and 4D5 exhibited GI values from 87% to 90% (Table S1). Furthermore, because the human-macaque chimeric mAbs contain the human IgG constant region, the overall human homology of the most chimeric mAbs was greater than 97% (Table S2). Therefore, it is reasonable to describe these human-macaque chimeric mAbs as “human-like” because they are highly homologous to human antibodies.

### Human-macaque chimeric mAbs protect mice after infection with H5N1 influenza virus

**Figure 4:**
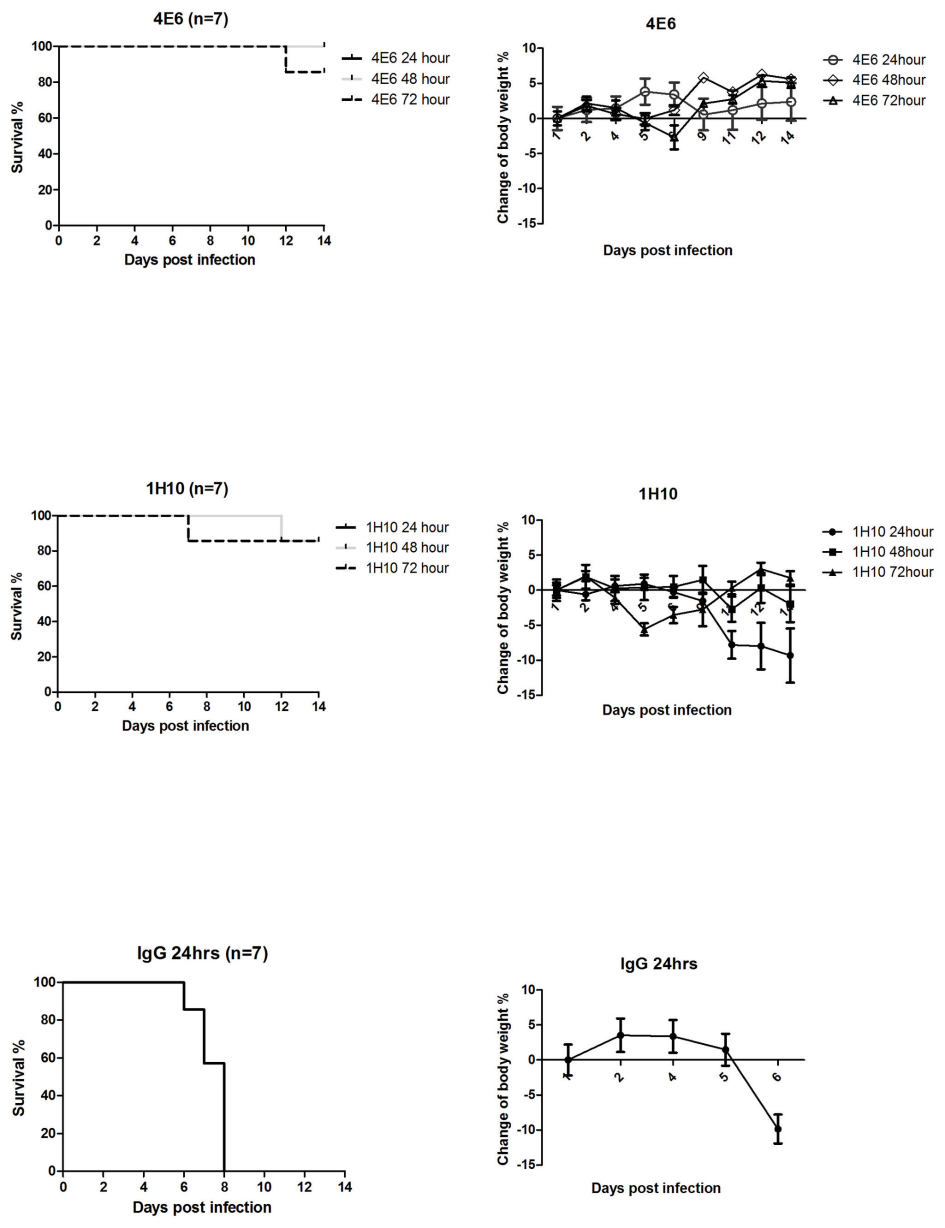
Therapeutic efficacy of mAb 4E6 and 1H10 in a mouse model of H5N1 virus A/Vietnam/1194/04 infection. (A) The Kaplan-Meier survival curve. BALB/c mice (n=7 per group in two separate experiments) were infected intranasally with 10 LD_50_ of A/Vietnam/1194/04 and at 24, 48, or 72 hours, were treated with a single intraperitoneal injection of mAb 4E6 or 1H10 at 10 mg/kg body weight. The control group received IgG at 10 mg/kg body weight at 24 hours post-infection. (B) Percentage weight change in mice treated with mAb 4E6 or 1H10 at different time points post-infection. The mice (n=7) were infected via intranasal inoculation with 10 LD50 of A/Vietnam/1203/4, followed by a single intraperitoneal injection of mAbs at 24, 48, or 72 hours post-infection, and their weights were monitored for 14 days. The mice injected with an irrelevant IgG at 24 hours post-infection were used as the control group.

**Figure 5:**
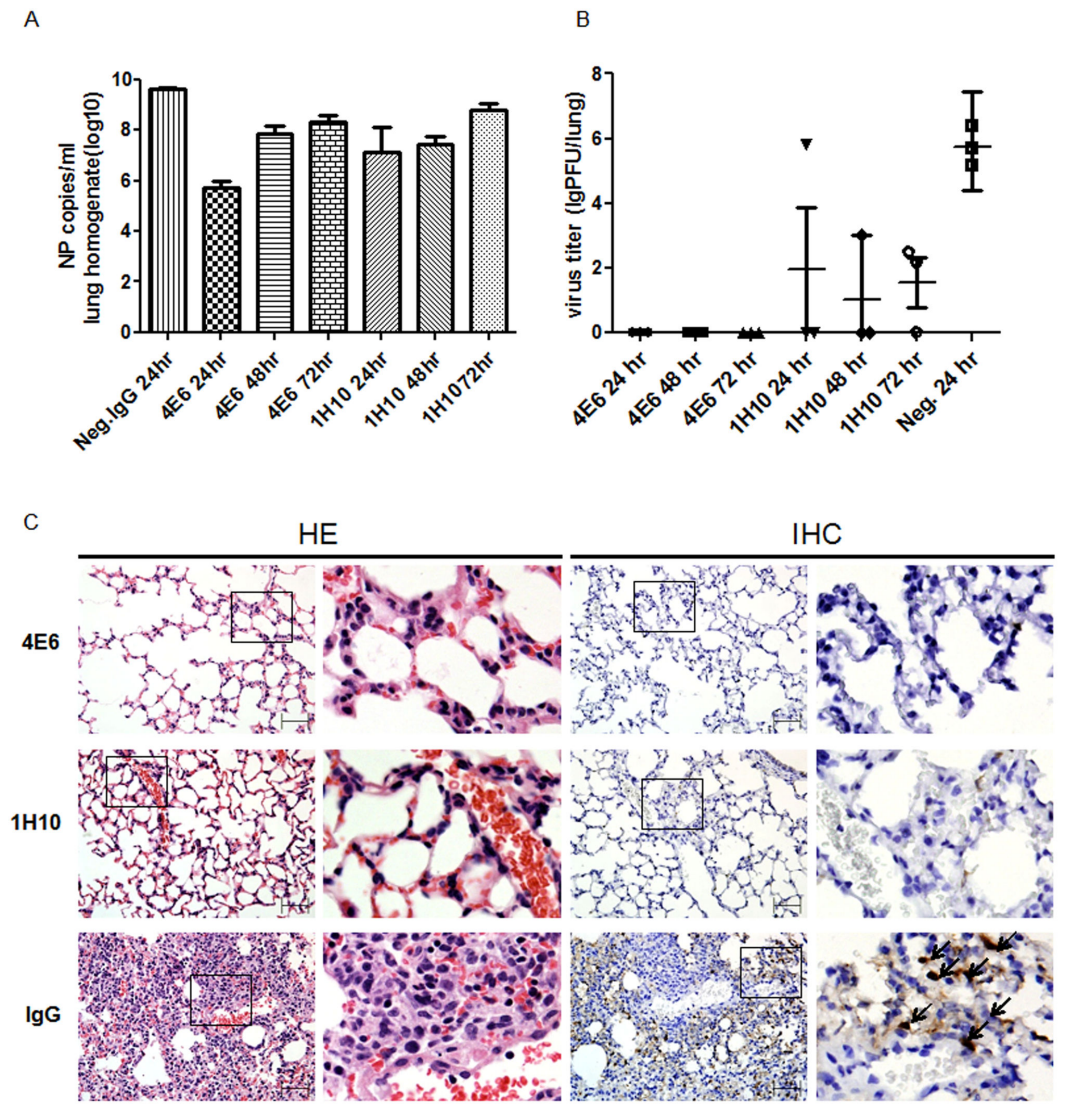
Analysis of lungs from mice treated with mAb 4E6 and 1H10 after infection with H5N1 virus A/Vietnam/1194/04. (A) Viral copy number in the lungs at day 6 post-infection was determined using Q-PCR. The results are expressed as the mean ± SEM. (B) Virus titer in the lungs at day 6 post-infection was measured using a plaque formation assay in MDCK cells. The results are expressed as the mean ± SEM. (C) Histopathology in pulmonary tissue (40x magnification). 1) Mouse treated with mAb 4E6 at 24 hours post-infection. 2) Mouse treated with mAb 1H10 at 24 hours post-infection. 3) Mice injected with an irrelevant IgG at 24 hours post-infection were used as the control. HE: H&E staining. IHC: immunohistochemical staining using a mAb against influenza nucleoprotein.

To evaluate whether these human-like mAbs confer protection *in vivo*, we investigated the therapeutic efficacy of mAbs 4E6 and 1H10 in BALB/c mice infected with a lethal dose of the H5N1 virus. These mAbs exhibit different binding kinetics to the HA protein and neutralize H5N1 via different mechanisms. The mice were first infected intranasally with 10 LD_50_ of the H5N1 virus A/Vietnam/1194/04. At 24 hours, 48 hours, or 72 hours post-infection, the mice were administered a single intraperitoneal injection of 4E6 or 1H10 at 10 mg/kg body weight. The mice that received the control IgG1 at 24 hours post-infection succumbed to infection (Figure 4A). In contrast, 100% of the mice that received 4E6 at 24 hours or 48 hours post-infection were protected, and there was 88% protection in the mice treated at 72 hours post-infection. Moreover, the mice treated with 4E6 did not exhibit a significant decrease in body weight because of the H5N1 infection (Figure 4B). The Q-PCR analysis of the lung tissues demonstrated that the mice treated with 4E6 exhibited up to 4 logs reduction in viral genome copy number compared with the control IgG-treated group (Figure 5A). Strikingly, none of the mice treated with mAb 4E6 had detectable live virus in their lungs at 6 days post-infection. In comparison, the mice that received the control IgG exhibited up to 1x10^6^ pfu of live virus per lung, whereas the mAb 1H10-treated mice had detectable but reduced (4-5 logs reduction) live viruses in their lungs (Figure 5B). This result suggested that mAb 4E6 exhibited an extremely potent neutralizing activity that completely inhibited the H5N1 virus. The histopathological examination demonstrated that there were no visible pathological changes in the lungs from the mice treated with 4E6 at 24 hours post-infection, whereas the control group that received an irrelevant IgG exhibited severe alveolar damage and interstitial inflammatory infiltration. The immunohistochemical staining of the lung cryostat sections with a mAb against the influenza nucleoprotein (NP) demonstrated the strong expression of NP in the cytoplasm of the pulmonary alveolar epithelial cells in the mice that received the control IgG but not in the mice treated with 4E6 (Figure 5C). The mAb 1H10, although not as impressive as mAb 4E6, also demonstrated a high potency for protecting the mice and for viral load reduction in the lungs. 1H10 conferred 88% protection in the mice treated at 24, 48 or 72 hours after the lethal infection. Taken together, the data suggested that mAb 1H10 exhibits *in vivo* therapeutic efficacy against the H5N1 virus, similar to several reported mAbs, whereas mAb 4E6 represents a unique mAb with superior potency for inhibiting the H5N1 virus. 

## Discussion

**Figure 6:**
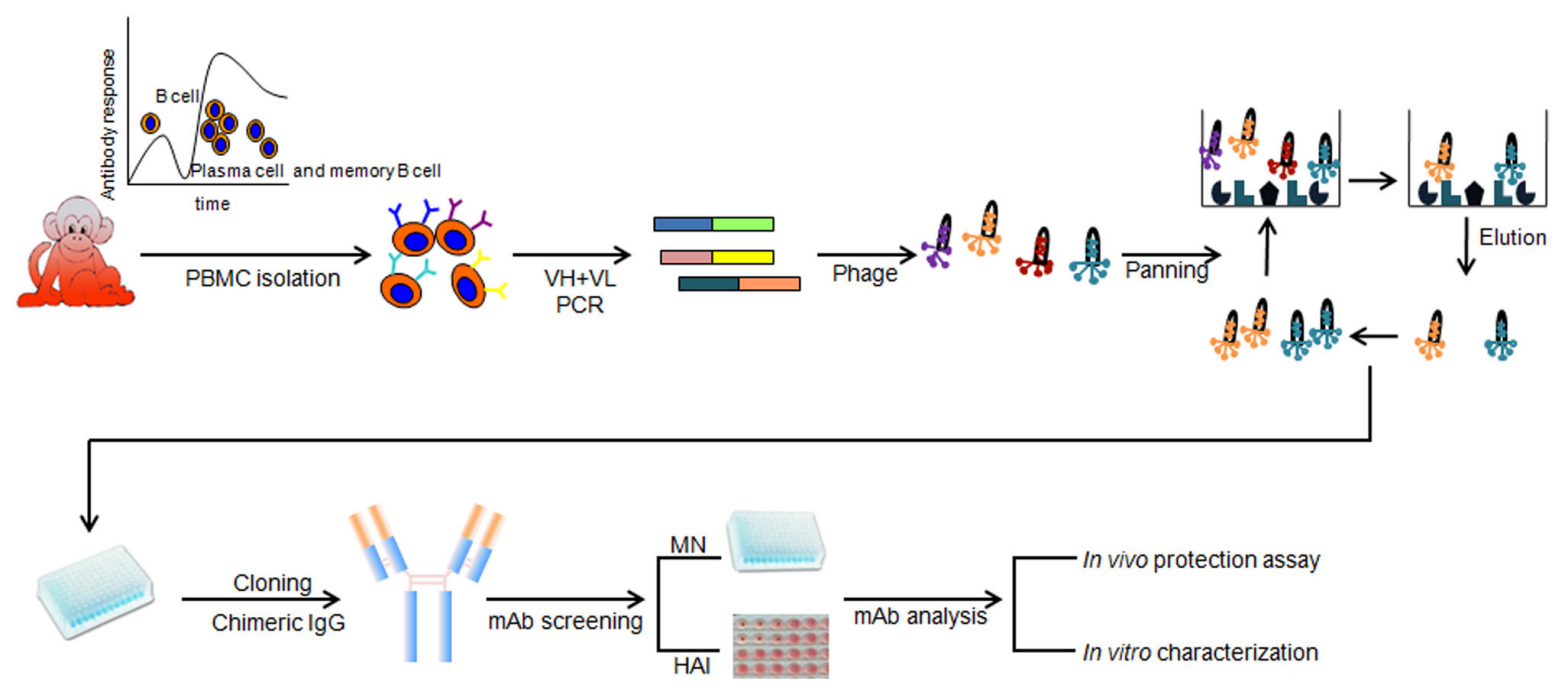
Schematic presentation depicting the method and process to generate human-like neutralizing mAbs through immunization of rhesus macaques.

In this proof-of-concept study, we generated human-macaque chimeric mAbs that exhibited high homology to human antibodies, high affinity and potent neutralizing activities. These human-like mAbs represent a potential solution to meet urgent needs during outbreaks of new influenza pandemics and other virulent infectious diseases. The human-like mAbs are a good alternative in the event of a virulent infectious outbreak, especially if there are no human survivors from the infection or ethical and regulatory issues arise that prevent the immunization of individuals with an antigen or a pathogen. The strategy and overall process we used in this study, i.e. immunizing rhesus macaques using adenoviral vectors carrying a synthetic gene encoding the antigen, followed by the rapid selection of mAbs using an immune-focused antibody phage display library or other mAb cloning technologies, will be of great practical use and significance. The overall process could be completed in approximately two months and entails the following five major steps (Figure 6): 1) Immunization of rhesus macaques at day 0; 2) Collection of PBMCs as early as day 14, or day 21 if a booster immunization is necessary; 3) Construction of an immunized antibody phage display library and selection of neutralizing clones, which could be completed in approximately one week; 4) Cloning the various regions of the macaque antibody into an expression plasmid containing the human immunoglobulin constant regions, which could be completed in a number of days; and 5) Production and characterization of chimeric mAbs, which could take weeks to months. However, it is important to understand that if a mAb is to be tested in humans, preclinical studies, such as safety and toxicity tests including screening against self-antigens, will be needed.

There is a unique advantage to generating antigen-specific neutralizing antibodies for therapeutic and prophylactic use through the immunization of rhesus macaques. Compared with mice and other animal species, the homology of the rhesus and human immunoglobulin genes is high. A recent analysis confirmed that the average homology between the corresponding rhesus VH and human VH genes is approximately 92% [14]. In our study, generating a chimeric antibody containing macaque VH and VL combined with human immunoglobulin constant regions, increased the degree of humanness in the full-length antibody to greater than 97%, which is much higher than human-mouse chimeric antibodies. The several human-mouse chimeric antibodies on the market contain murine-derived VH and VL and human-derived constant regions, such as infliximab/Remicade (anti-TNFα), rituximab/Rituxan (anti-CD20), cetuximab/Erbitux (anti-EGFR), and basiliximab/Simulect (anti-IL2R). However, approximately 40% of human-mouse chimeric antibodies encounter significant human anti-antibody responses in patients, compared to 9% for humanized antibodies [33]. The human-macaque chimeric antibodies are more suitable for application in humans because they are highly homologous to human antibodies and are less likely to induce human anti-chimeric antibodies than the human-mouse chimeric antibodies. This concept is supported by reports of at least two human-macaque chimeric mAbs, galiximab (IDEC-114, anti-CD80) and lumiliximab (IDEC-152, anti-CD23), in which the VH and VL from cynomolgous macaques were fused with human constant regions. In phase I and II clinical studies, no human anti-galiximab or anti-lumiliximab antibodies were detected in humans [34,35]. Finally, if needed, it would be easier to change a small number of amino acids in the framework region of the macaque VH and VL regions to produce complete germline humanization [36]. However, we believe that this change would likely be unnecessary because the framework region in our mAbs were within the range of humanness when compared with the human counterparts.

Using the H5N1 influenza virus as a model, we demonstrated that human-like mAbs, exhibiting high affinities at sub-picomolar to nanomolar levels and high neutralizing potencies, could be generated using a simple method. Based on published information regarding human mAbs against the H5N1 virus A/Vietnam/1194/04, compared with mAb F10 (K_D_ =130 pM), the mAb 4E6 exhibited the highest affinity (K_D_ =22 pM) for H5N1 VN04HA [9]. The 4E6 mAb demonstrated a slow dissociation rate to the HA protein (6.14x10^-5^/s^-1^). Interestingly, 4E6 contains a somewhat longer CDR3 region, consisting of 19 amino acids, than the CDR3 reported for most human mAbs to influenza virus HA, which contain 12 to 17 amino acids [3,9]. We compared the 4E6 data with published data from several mAbs of human origin that have been tested in mouse models. Corti et al. described two heterosubtypic mAbs, FE43 and FE17; however, these antibodies were tested only in prophylactic mouse models in which a survival rate of 20% and 100%, respectively, was observed when used at 2.5 mg/kg body weight [10]. Simmons et al. identified three mAbs, FLA3.14, FLD21.14, and FLD20.19, which demonstrated 80%-100% therapeutic protection in mice infected with 5 LD50 H5N1 VN/04 when administered at the higher dosage of 50 mg/kg body weight [11]. Sui et al. identified several broad-spectrum mAbs, D8, F10 and A66, exhibiting affinities of 148 pM, 130 pM and 233 pM, respectively, to H5N1 VN04HA [9]. The F10 and A66 mAbs demonstrated 80-100% therapeutic protection when administered at 15 mg/kg body weight, whereas D8 was poorly effective in the therapeutic model. However, in these mAb-treated mice, between 1x10^3^ and 1x10^6^ pfu of live virus was detected per gram of lung tissue in the treated groups [9]. De Marco et al. identified a non-VH1-69-derived human mAb PN-SIA49 that recognizes a highly conserved epitope in the stem region of HA and exhibits a broad spectrum, neutralizing H1, H2 and H5 subtypes [37]. However, at a 10 mg/kg dose administered at 24 hours post-infection, SIA49 afforded only 67% protection in mice infected with 3 LD50 of H5N1 A/VN/1203/04. Comparing these reported mAbs with 4E6 demonstrated that mAb 4E6 is at least among the best of the mAbs reported in the literature that exhibit *in vivo* therapeutic efficacy against the H5N1 virus. In addition to the indistinguishable difference in the degree of humanness between 4E6 and mAbs of human origin, 4E6 conferred 100% protection in mice at 24 and 48 hours after infection and 88% protection in mice at 72 hours after infection with 10 LD50 of H5N1 A/VN/1194/04. Furthermore, a single treatment completely inactivated the H5N1 VN/04 virus in all the treated groups. We speculate that the extremely slow dissociation rate (6.14x10^-5^/s^-1^) and the HA binding epitope of 4E6 may play a role in the impressive potency of this mAb. Based on the data from the escape mutant, the 4E6 bound to a conserved site on the globular head of HA, and amino acids D183 and K/R198 are present in most H5N1 viruses. A single mutation at D183 or K/R189 in HA did not diminish the binding of 4E6. Therefore, a virus would need two mutations, at both D183 and K189, to escape neutralization by 4E6.

Taken together, our study demonstrated that for preparedness against outbreaks of virulent infectious diseases in the future, human-like neutralizing mAbs exhibiting high affinities and high potencies could be generated readily from an immune-focused antibody phage display library derived from rhesus macaques immunized with target antigens. Alternatively, mAbs could be selected from single B cells or antibody-secreting plasma cells (ASC). Although it has been reported that mAbs can be cloned through the RT-PCR of immunoglobulin genes from single B cells or ASC cells isolated from individuals infected with influenza virus or who received influenza vaccines [4,8], the technical requirement for the B cell manipulation requires sophisticated expertise and laboratory equipment, which may not be readily available, especially in developing countries.

In summary, we have demonstrated a practical option for obtaining high-affinity, high-potency neutralizing mAbs for urgent needs, which may be advantageous in terms of effectiveness, speed, simplicity, and practicality. The mAbs described in this study have the potential to be readily available for human application, especially in the midst of outbreaks of virulent infectious disease. Our study warrants further investigation for the development of protection against outbreaks of new influenza pandemics and other emerging infectious diseases.

## Materials and Methods

### Construction of replication-incompetent adenovirus carrying codon-optimized genes encoding HAs

Recombinant replication-incompetent E1-deleted serotype 5 adenoviruses carrying the HA gene (Ad5-HA) from various H5N1 viruses were generated based on homologous recombination as described previously [19]. The expression cassette, containing the HA gene under the control of CMV promoter and bovine growth hormone (BGH) poly(A) signal, was cloned into the adenoviral E1 region. The HA sequences from influenza virus H5N1 were obtained from the Influenza Sequence Database (www.flu.lanl.gov). The HA genes were optimized according to preferred codon usage for optimal expression in mammalian cells and were synthesized chemically. The recombinant adenoviruses were produced in 293 cells and were purified by cesium chloride gradient centrifugation. The viral particle number was determined by measuring the absorbance at 260 nm. The Ad5-HA viral genome was extracted using proteinase K digestion and restriction enzyme digestion and PCR was used to confirm the genomic integrity. The Ad5-HA-mediated expression of HA proteins was confirmed by Western blot analysis using chicken serum against HA.

### Influenza Viruses

The HA genes of the A/Vietnam/1194/04, A/Hong Kong/482/97 and A/ZheJiang/16/06 (H5N1) influenza viruses were modified by deleting the polybasic amino acids in the HA. These mutated HA sequences were used to generate recombinant viruses with the H1N1 influenza virus A/Puerto Rico/8/34 (PR8) background. The recombinant influenza viruses were generated using a 12-plasmid-based reverse genetics system as described previously [38]. At forty-eight hours after transfection, the supernatant from the transfected cells was collected and was inoculated into the allantoic cavities of 10-day-old embryonated chicken eggs for virus propagation. The correct sequences of the recombinant viruses were confirmed by nucleotide sequence analysis.

### Immunization of rhesus macaques

The two male Chinese rhesus macaques (No. 04068 and 050105), aged about 5 years, were immunized with a mixture of the three AdH5-HA viruses (1x10^11^ particles of each virus) via intramuscular injection and were boosted at 4 weeks and 8 weeks. One micro liter of blood from each rhesus macaque was collected every two weeks for plasma antibody examination.

### Ethics statement

The animals were housed and handled in accordance with the guidelines set by the Association for the Assessment and Accreditation of Laboratory Animal Care. The study protocol was approved by the GIBH Institutional Animal Care and Use Committee (Protocol number 2011038). All steps taken to ameliorate the welfare and to avoid the suffering of the rhesus monkeys were in accordance with the recommendations of the “Weatherall report for the use of non-human primates". The animals were housed in adjoining individual primate cages allowing social interactions, under conditions of controlled humidity, temperature and light (12-hour light/12-hour dark cycles). Food and water were available ad libitum. The animals were monitored daily and fed commercial monkey chow, treats and fruit twice daily by trained personnel. Environmental enrichment consisted of commercial toys. All procedures, including the immunizations and blood draws, were performed under sedation by trained personnel and under the supervision of veterinarians. The rhesus monkeys were in good condition and were re-housed after the experiments.

### Construction and screening of the scFv phage library

The primers used to amplify the rhesus macaque heavy-chain and light-chain variable regions were designed based on previous publications of the rhesus macaque immunoglobulin gene sequences [22-31]. The sequences were grouped into the 7V_H_, 6V_*κ*_, 11V_*λ*_, and 7J_H_ families in accordance with the IMGT nomenclature. Based on the nucleotide sequence alignment of the individual families, we designed a set of primers targeting the conserved motifs in framework region 1 and the joining and constant regions of the H- and L-chain genes. To design the reverse primers for the heavy chain, we analyzed separately the 5’ sequences of IGHV1, IGHV2, IGHV3, IGHV4, IGHV5, IGHV6 and IGHV7. For the 3’ forward primers for the heavy chain, we focused on the 3’ sequences of six IGHJ genes. Based on the reported macaque light-chain sequence, we designed primers for 3 IGKV subgroups and 4 IGLV subgroups. The primer sequences were degenerate at a number of positions to maximize the amplification efficiency while minimizing the creation of stop codons. Blood samples (10 ml) were collected from each rhesus macaque. The peripheral blood B lymphocytes were isolated using OptiPrep (Axis-shield, Norway). The mRNA was extracted from the lymphocytes using a Qiagen Blood RNA kit (Qiagen) and was reverse-transcribed using primers that were specific to the rhesus macaque IgG, Igκ and Igλ constant regions [25-28]. The cloning of the VH and VL DNA fragments into the pCANTab 5E phage display vector was performed as described previously [39]. The library was panned against the recombinant HA protein of A/Vietnam/1194/04. After two rounds of phage panning, the individual clones from the enriched phage pools were analyzed using ELISA against influenza viruses. The positive clones were sequenced to determine the heavy- and light-chain sequences. The sequences were analyzed using the International ImMunoGeneTics information system^®^ (IMGT) (http://imgt.cines.fr) and were compared with the sequences of human immunoglobulin genes.

### Construction and Expression of the full-length human-macaque chimeric mAbs

The selected scFvs were converted into full-length antibodies by cloning the VH and VL sequences in-frame into the human IgG1 constant regions, HC and LC, in expression plasmids that expressed the light chain and the heavy chain separately. The expression vectors were modified based on the backbone of the pGI-neo vector. Equal molar amounts of heavy-chain plasmid and light-chain plasmid were co-transfected into 293T cells for transient expression, and the supernatants were harvested four days after transfection. The IgG was purified by affinity chromatography using protein A.

### Immunofluorescence analysis of mAb binding to virus-infected cells

The MDCK cells were grown as monolayers on glass slides in 6-well plates and infected with recombinant H5N1 viruses at MOI of 1.0. At 18 hours post-infection, the cells were fixed using freshly prepared 4% paraformaldehyde for 15 minutes at 4°C and were permeabilized using PBS containing 0.05% Triton X-100 for 5 min at room temperature. The cells were blocked in PBS containing 5% BSA for 1 hour and were incubated with the mouse anti-NP primary antibody and our mAbs in PBS containing 5% BSA for 1 hour. The bound antibodies were detected using Cy3-conjugated anti-mouse IgG secondary antibody and FITC-conjugated anti-human IgG secondary antibody under confocal microscopy (Leica, Germany).

### Micro-neutralization (MN) assay

The neutralizing antibody titers were determined essentially as described previously [40]. In brief, 50 µl of influenza virus at 100 TCID(50) was incubated with 50 µl of a two-fold dilution of the purified IgG for 2 hours at 37°C in a 96-well plate containing an MDCK cell monolayer. After incubation, the virus-antibody samples were removed from the wells, and the cells were incubated at 37°C for 2 days in minimal essential medium containing bovine albumin. The neutralization titer was defined as the highest dilution of antibody.

### Hemagglutination inhibition (HAI) assay

The HAI assays were performed using the standard methods described previously [41]. Briefly, 25 µl of each influenza virus strain at a HA titer of 8 HA units was mixed with 25 µl of a two-fold dilution of the purified IgG in PBS in v-bottomed 96-well plates. After 20 minutes incubation at room temperature, 50 µl of 0.5% chicken erythrocytes was added to the mixtures. The plates were maintained at room temperature for 30 minutes. The HAI titer was defined as the highest dilution of the plasma or the purified mAbs that inhibited hemagglutination.

### Surface Plasmon Resonance (SPR) Assay

The kinetic analyses of the mAb binding to the recombinant H5-VN04 monomers were performed on a Biacore 3000 device (Biacore, Sweden) at 25°C. Anti-human IgG Fc antibody (Biacore) was covalently attached to the individual flow cell surfaces of a CM5 sensor chip through amine coupling using the amine coupling kit (Biacore). The HA mAbs were captured by the anti-human Fc surface. The HA protein was injected over each flow cell in HBS-EP buffer at a flow rate of 30 µl/min at concentrations 20, 10, 5, 2.5, 1.25, and 0.625 nM. The experiments included an additional anti-human IgG Fc antibody control surface to account for changes in the refractive index of the buffer and to test for potential nonspecific interactions between the HA and the anti-human IgG Fc. The resonance units (RU) reflected the concentration of the antibody-antigen complex. The association rates (*ka*), dissociation rates (*kd*), and affinity constants (*K*_*D*_) were calculated using the Biacore 3000 evaluation software using a 1:1 Langmuir binding model with mass transfer and the global analysis method. The quality of each fit was based on the agreement between the experimental data and the calculated fits, in which the Chi^2^ values were below 2.0.

### Generation of neutralizing antibody escape mutant

The escape mutants were selected as described previously, with some modifications [42]. In brief, 100 µl of 150 pfu of r04VNHA-PR8 and 100 µl of 32 HIU (Hemagglutination Inhibition Unit) 4E6 were incubated at 25°C for 1 h and then at 4°C overnight. The virus-antibody mixtures were inoculated into 10-day-old embryonated chicken eggs. The allantoic fluid was harvested after 48 h incubation at 35°C and the plaques were purified. Single isolated plaques were selected and were amplified in 10-day-old embryonated chicken eggs in the presence of the 4E6 antibody. The escape mutants were harvested and were tested using the HAI assay and the MN assay, followed by genomic sequencing and Western blot analysis.

### *In vivo* protection experiment

The therapeutic protection experiment was performed as previously reported [43]. The female BALB/c mice (6-8 weeks old) were housed in an accredited biosafety level 3 (BSL3) animal facility in accordance with the protocols approved by the HKU Animal Care and Use Committee. Any animal that lost 25% of its starting body weight at the end of the experiment was sacrificed via CO_2_ inhalation. The inoculation of the mice and the tissue harvests were performed in a biosafety cabinet by personnel wearing powered air purifying respirators. For the therapeutic efficacy study, 10 mice per group were inoculated intranasally with 10LD50 of H5N1 virus A/Vietnam/1194/04 in a volume of 50 µl. At various times after the infection, a single injection of mAb was administered via intraperitoneal injection in a volume of 0.5 ml. A human IgG was used as the negative control and was injected at 24 hours after infection. To determine the viral load in the infected lungs, 3 mice from each group were sacrificed on day 6 post-infection. The right lungs were collected and homogenized in 1 ml cold MEM. The virus titers from the clarified lung homogenates were determined using a plaque assay on MDCK cells and real-time PCR detection. The left lungs were fixed for the histopathological analysis. The remaining 7 mice in each group were monitored and weighed daily for 14 days after the infection.

### Histopathological Analysis

The lungs were fixed immediately in 10% buffered formalin and were embedded in paraffin wax. The 4–6-µm thick sections were mounted on the slides. Following H&E staining, the histopathological changes were analyzed under a light microscope as described. The lung sections were stained as described using an anti-influenza nucleoprotein mAb (HB65; ATCC) at a 1:5,000 dilution, goat anti-mouse IgG, H and L chain-specific biotin conjugates (Calbiochem) at a 1:2,000 dilution, and streptavidin/peroxidase complex reagent (Vector Laboratories).

## Supporting Information Legends

Figure S1 mAb 4E6 binds to a conserved epitope on HA globular head. (A) Western blot analysis of purified virions of r04VNHA-PR8 and the escape mutant (D183N, K189E). The samples were blotted against 4E6 and an anti-NP monoclonal antibody, respectively. (B) 3D structure of HA protein. The 4E6 binding sites on HA are highlighted in red (D183) and yellow (189). (C) Protein sequence alignment for residues 160 to 200 (H5 numbering) in HA from the H5N1 viruses used in this study (clades 0, 1, and 2) and other clades (3, 4, 5, 6, 7, 8, and 9). (D) Protein sequence alignment for residues 160 to 200 (H5 numbering) in the HA from the wild-type H5N1 viruses tested for *in vitro* assays in our study and our escape mutant.
(TIF)

Table S1 Analysis of rhesus humanness based on germline index (GI). GI = (number of identity Framework amino acid) / (total number of Framework amino acid) [29]. F10 and CR6261 are fully human mAbs against influenza viruses reported in previously literature [9,12]. GI value was achieved by online analysis at http://www.imgt.org/3Dstructure-DB/cgi/DomainGapAlign.cgi.
(DOCX)

Table S2 Alignment rhesus macaque heavy chain and light chain variable regions with closest human germline genes. The amino acid sequences of heavy chain and light chain variable regions were compared with their closest human germline counterparts. The full-length human-macaque chimeric mAbs were also compared.
(DOCX)
