# Supplementary figures and images for: Rapid Generation of Human-Like Neutralizing Monoclonal Antibodies in Urgent Preparedness for Influenza Pandemics and Virulent Infectious Diseases
Source: PLoS One. 2013 Jun 18;8(6):e66276. doi: 10.1371/journal.pone.0066276 (PMC3688872; doi:10.1371/journal.pone.0066276)

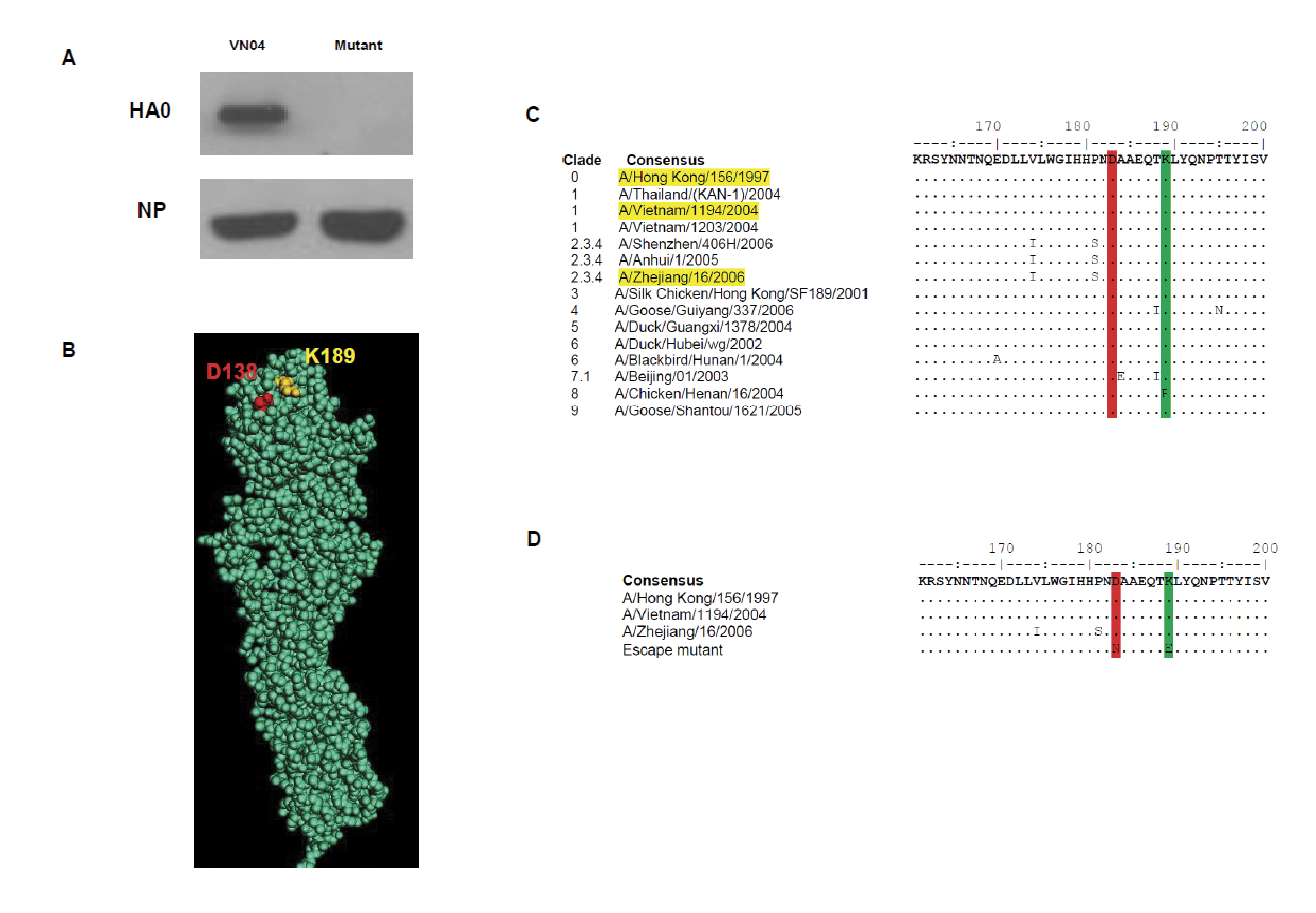

Supplement: Figure S1 — (A) Western blot analysis of purified virions of r04VNHA-PR8 and the escape mutant (D183N, K189E). The samples were blotted against 4E6 and an anti-NP monoclonal antibody, respectively. (B) 3D structure of HA protein. The 4E6 binding sites on HA are highlighted in red (D183) and yellow (189). (C) Protein sequence alignment for residues 160 to 200 (H5 numbering) in HA from the H5N1 viruses used in this study (clades 0, 1, and 2) and other clades (3, 4, 5, 6, 7, 8, and 9). (D) Protein sequence alignment for residues 160 to 200 (H5 numbering) in the HA from the wild-type H5N1 viruses tested for in vitro assays in our study and our escape mutant. (TIF) [file pone.0066276.s001.tif]
