# Supplementary material for: Rapid Generation of Human-Like Neutralizing Monoclonal Antibodies in Urgent Preparedness for Influenza Pandemics and Virulent Infectious Diseases
Source: PLoS One. 2013 Jun 18;8(6):e66276. doi: 10.1371/journal.pone.0066276 (PMC3688872; doi:10.1371/journal.pone.0066276)
Supplement: Table S1 — GI = (number of identity Framework amino acid) / (total number of Framework amino acid) [29]. F10 and CR6261 are fully human mAbs against influenza viruses reported in previously literature [9,12]. GI value was achieved by online analysis at http://www.imgt.org/3Dstructure-DB/cgi/DomainGapAlign.cgi. (DOCX) [file pone.0066276.s002.docx]

| Clone | HC | | LC | |
| --- | --- | --- | --- | --- |
|  | GI | Resource | GI | Resource |
| 4E6 | 86.81% | IGHV4-28*01 | 88.86% | IGLV1-40*01 |
| 4D5 | 90% | IGHV4-b*01 | 86.36% | IGLV3-19*01 |
| 1H10 | 90% | IGHV1-69*13 | 89.7% | IGLV1-40*03 |
| CR6261^12^ | 84% | IGHV1-69 | NA | NA |
| F10^9^ | 91.1% | IGHV1-69*01 | 95.5% | IGLV10-54 |
| D8^9^ | 92.75% | IGHV1-69*01 | 89.66% | [IGLV2-8*01](http://www.imgt.org/IMGT_GENE-DB/GENElect?query=9+IGLV2-8*01&species=Homo+sapiens) |
| A66^9^ | 88.4% | IGHV1-69*01 | 96.2% | IGKV3D-11*01 |
