# Supplementary material for: Rapid Generation of Human-Like Neutralizing Monoclonal Antibodies in Urgent Preparedness for Influenza Pandemics and Virulent Infectious Diseases
Source: PLoS One. 2013 Jun 18;8(6):e66276. doi: 10.1371/journal.pone.0066276 (PMC3688872; doi:10.1371/journal.pone.0066276)
Supplement: Table S2 — The amino acid sequences of heavy chain and light chain variable regions were compared with their closest human germline counterparts. The full-length human-macaque chimeric mAbs were also compared. (DOCX) [file pone.0066276.s003.docx]

|  | Heavy chain | | | Light chain | | |
| --- | --- | --- | --- | --- | --- | --- |
|  | Closest human IGHV gene | Framework identity | Full-length identity | Closest human IGLV gene | Framework identity | Full-length identity |
| 4E6 | IGHV4-28*01 | 86.8% | 96.9% | IGLV1-40*01 | 88.9% | 97.4% |
| 4D5 | IGHV4-b*01 | 90.0% | 97.7% | IGLV3-19*01 | 86.4% | 96.8% |
| 1H10 | IGHV1-69*13 | 90.0% | 97.7% | IGLV1-40*03 | 89.7% | 96.9% |
